# Primary Clear Cell Carcinoma of the Pancreas: A Systematic Review

**DOI:** 10.7759/cureus.15668

**Published:** 2021-06-15

**Authors:** Toufic Tannous, Audrik L Perez Rodriguez, Andrew W Mak, Karim Tannous, Matthew Keating

**Affiliations:** 1 Internal Medicine, Roger Williams Medical Center/Boston University, Providence, USA; 2 Hematology and Medical Oncology, Roger Williams Medical Center, Providence, USA; 3 Division of Hematology/Oncology, Roger Williams Medical Center/Boston University, Providence, USA; 4 Faculty of Medicine, University of Balamand, Faculty of Medicine, Tripoli, LBN; 5 Hematology and Medical Oncology, University of California Irvine, Irvine, USA

**Keywords:** ductal pancreatic carcinoma, primary clear cell carcinoma of the pancreas, clear cell cancer, molecular biomarker, carcinoma pancreas

## Abstract

Over the years, the world has witnessed many advances in diagnosing and treating multiple types of cancers. These breakthroughs have revolutionized the understanding of the molecular drive behind these neoplasms, leading to tangible therapeutic evolution and promising prognostic implications. However, pancreatic cancer remains a highly lethal disease. With recent discoveries, modern medicine has been able to delineate histopathologic subtypes of pancreatic cancer in hopes of improved diagnosis and treatment to improve survival. A once vague entity, clear cell adenocarcinoma of the pancreas, in particular, has been better characterized on a histopathological and molecular level over the past two decades. With novel technological support, this disease has become less inconspicuous, and more researchers have reported its occurrence. Its diagnosis relies heavily on a mix of histological and immunohistochemical clues such as a clear cell cytoplasm and positivity for cytokeratins and other markers. However, new molecular markers, such as hepatocyte nuclear factor 1 beta, have been associated with this entity and may aid in further diagnostic and therapeutic strategies. This review article aims to portray how the identification and description of clear cell adenocarcinoma of the pancreas have evolved over the past few decades and how this may impact future treatment strategies.

## Introduction and background

There exists great interest in the early detection of pancreatic malignancies, especially pancreatic ductal adenocarcinoma (PDAC). Thus far, efforts have been geared towards investigating biological factors using novel technologies to gain more insight into disease characteristics [1]. PDAC remains extremely lethal, and, unfortunately, its management has not changed substantially over the past few decades despite this area being a highly active field for ongoing research with a special focus on methods for early detection and improving prognosis. All these factors culminate into a projected trajectory that PDAC will be the leading cause of cancer-related deaths in the near future [2-3]. Risk factors are rather non-specific such as age, obesity, and smoking. Therefore, selecting the appropriate individuals for screening remains challenging and arduous [4].

With the advent of and advancements in next-generation sequencing, multiple genetic mutations have been associated with neoplastic transformation, such as KRAS, a signaling protein involved in the epidermal growth factor receptor formation pathway and found to be defective in over 90% of PDAC tumors. Several other mutations have been identified as well, including SMAD4/DPC4, TP53, and CDKN2A [5]. Not only have these molecular discoveries paved the way for developing novel targeted therapies, but they have also been integrated into the classification of neoplasia and refinement of treatment modalities. PDAC is subcategorized into four molecular groups, each with its own histopathological and clinical characteristics, which ultimately facilitate prognostication and therapeutic selection. The squamous subclass harbors TP53 and KDM6A mutations; the pancreatic progenitor tumors express genes required for pancreatic development, namely, FOX A2/3, PDX, MNX1; the aberrantly differentiated endocrine exocrine subclass has certain upregulated genetic expressions that activate KRAS; and the immunogenic subclass has upregulated genes required for the acquired immune suppression pathway [6-7]. This subclassification is not entirely agreed upon by experts and practitioners and remains an evolving field. Moreover, fully understanding a certain type of cancer starts with elucidating its histological characteristics, which then expose its molecular roots.

In this review, we take a closer look at clear cell carcinoma of the pancreas (CCCP), one of the most inconspicuous morphological subtypes of PDAC. Our aim is to further describe this entity and synthesize the findings from previously published case reports and studies over the past 40 years. Due to the rarity of CCCP, we believe that a review is vital to understand its genetic, molecular, clinical, and prognostic implications. We focus on describing the historical evolution in the understanding of this disease over the years.

## Review

Methods

**Figure 1:**
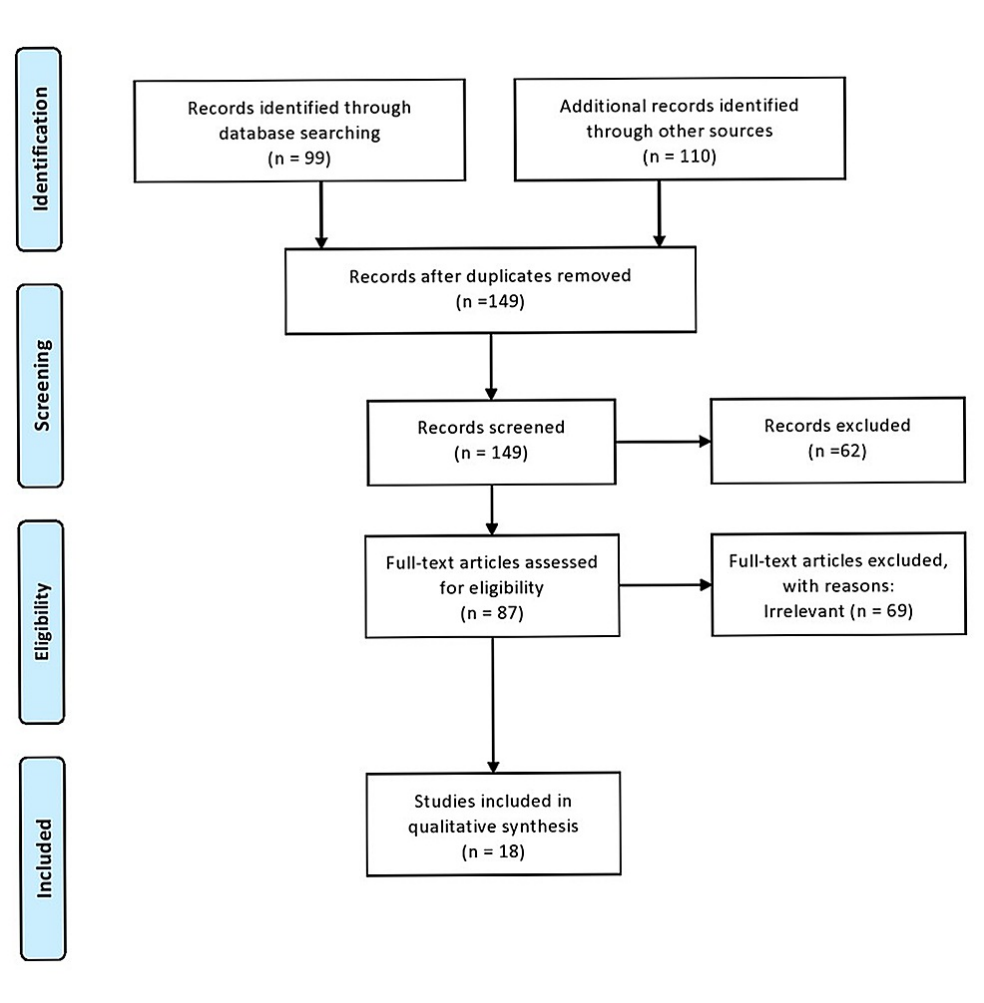
Preferred Reporting Items for Systematic Reviews and Meta-Analyses (PRISMA) flow diagram for our systematic review

Using the databases PubMed and Google Scholar, we attempted to collect all published case reports, case series, systematic reviews, and original research describing CCCP from the first publication in 1975 until the present year (2021). We focused on cases of clear cell pancreatic tumors of ductal origin that have been published in the English language. The search was conducted with the use of the appropriate keywords and medical subject headings (MeSH) terms. The keywords used to collect the relevant reports included “primary clear cell carcinoma of the pancreas.” Cases describing metastatic clear cell carcinomas to the pancreas, as well as clear cell “sugar” tumors of the pancreas and clear cell neuroendocrine tumors of the pancreas, were excluded. Data selection was performed autonomously by two authors (T.T and M.K) independently. In cases of disagreement, both authors thoroughly discussed the relevance of the article to our inclusion and exclusion criteria. EndNote 20 (Clarivate Analytics, Philadelphia, PA) was used to sort out the differences and ensure proper article citation. After collecting all the published case reports, case series, and reviews, we extracted all the relevant clinical data (including age, sex, risk factors, pathology, immunohistochemistry, treatment, and outcome). This systematic review was designed using the Preferred Reporting Items for Systematic Reviews and Meta-analysis (PRISMA) as detailed in Figure 1 [8].

Results

A total of 209 published articles were identified using the search engines PubMed and Google Scholar. After the removal of 60 duplicates, 149 articles remained. Based on the title and the content of their abstract, these articles were filtered, out of which 62 were excluded, and 87 full-text articles were assessed for eligibility. Sixty-nine (69) articles were subsequently removed, leaving a total of 18 eligible articles, of which 14 were case reports and four were systematic reviews.

Beginnings

In 1975, Cubilla and Fitzgerald described the different types of non-endocrine pancreatic cancer cases encountered at Memorial Hospital in New York City between 1949 and 1972 [9]. The purpose of their study was to categorize the morphological patterns of pancreatic cancer in order to better understand the clinical and prognostic implications. Out of 406 cases reviewed, they were able to stratify non-endocrine pancreatic tumors into the following two groups: benign epithelial non-endocrine neoplasms and malignant epithelial neoplasms. The latter was further divided into carcinomas, sarcomas, primary malignant, lymphomas, miscellaneous, and metastatic cancer. The carcinomas included multiple subcategories such as duct cell adenocarcinoma, giant cell carcinoma, microadenoma, adenosquamous carcinoma, mucinous adenocarcinoma, anaplastic carcinoma, cystadenocarcinoma, acinar cell carcinoma, carcinoma in childhood, and unclassified. Duct cell adenocarcinoma comprised the bulk of the cases (76%), and the unclassified cases were a distant second (7%) in terms of frequency. They were also able to isolate and identify six cases of anaplastic carcinomas, which were also referred to as indeterminate carcinomas manifesting as tumor cells arranged in chords or nests staining positive for mucin in half the cases.

In their 1980 publication, Cubilla and Fitzgerald further refined their classification system for non-endocrine pancreatic cancers based on their experience with the cases encountered at Memorial Hospital and cases described in the literature [10]. They defined four classes of primary non-endocrine pancreatic cancers based on 645 cases reviewed. The bulk of the cases were considered of ductal cell origin (further subdivided into seven categories), acinar cell origin, uncertain histogenesis, and connective tissue origin. As in their previous publication, anaplastic carcinoma cases were listed but were described in more detail. They noted that most neoplasms were diagnosed at autopsy and described the tumor cells as either large cell-like, lymphoma-like, or small-cell-like. One type displayed a clear cytoplasmic appearance that was positive for mucin and resembled the clear cell type found in renal and adrenal clear cell carcinomas. This was the first time CCCP was described.

In 1982, Urbanski and colleagues described the case of a man who presented to Toronto Western Hospital with abdominal pain, diarrhea, and facial flushing [11]. He was found to have metastatic deposits on the liver and was diagnosed with a cancer of unknown origin. Unfortunately, the patient died shortly thereafter. During his autopsy, a pancreatic body lesion was discovered that consisted of numerous giant eosinophilic and spindle cells on microscopic analysis. Interspersed within these cells were large cells with clear cytoplasm and a hyperchromatic prominent nucleus. The cytoplasm tested positive for mucin on mucopolysaccharide and Periodic Acid-Schiff (PAS) staining. The metastatic deposits on the liver and retroperitoneal lymph nodes were composed entirely of clear cells. The presence of such findings was quite surprising to the authors, as they had never encountered CCCP. However, they entertained the possibility of other types of clear cell carcinomas that were more common such as renal clear cell and adrenal clear cell. But the presence of mucin made them less likely, and the lack of glycogen and lipids excluded pulmonary, ovarian, and endometrial clear cell carcinomas. They eventually termed the case as a clear cell variant of the pleomorphic giant cell carcinoma.

In 1987, Kanai and colleagues reported the case of a 71-year-old Japanese man who presented with abdominal pain [12]. His laboratory workup revealed elevated carbohydrate antigen 19-9 (CA19-9) and carcinoembryonic antigen (CEA) levels. Abdominal computed tomography (CT) scan showed a pancreatic tumor and stomach lesions. He was diagnosed with a primary pancreatic malignancy and treated with Mitomycin C and Fluorouracil (5-FU), but due to his extensive disease burden and development of lymphangitic carcinomatosis in the lungs, he eventually succumbed to his malady. Upon autopsy, a 6x10 cm pancreatic mass was discovered, which encased the body and tail with extensive tumor deposits over the left kidney, gastric body, liver, and spleen. He was assumed to have metastatic gastric cancer until a microscopic examination was performed. The pancreatic tumor cells were round/oval with distinct cell borders. The presence of extracellular mucin was detected with the absence of a hobnail cellular arrangement. The cytoplasm was vacuolated and clear, staining weakly positive for PAS and negative for Sudan III stain. The varied between central and peripheral locations. The same examination was noted in all the other metastatic sites. Although the clinicians were perplexed at first, as clear cells of the pancreas were practically unheard of at the time, other pathologies were ruled out due to the tumor cells containing mucin and lacking glycogen and lipids. Here, the diagnosis was considered to be pure CCCP since the clear cells made up the entire cell histology.

Advancements in Immunohistochemistry

Advancements in immunohistochemistry (IHC) and electron microscopy provided more insight into the nature of CCCP. Luttges and colleagues described a 53-year-old man who presented with abdominal pain, elevated CA19-9, and CT findings of a pancreatic head tumor [13]. Microscopic examination of the tumor showed large cells with abundant and clear cytoplasm and large polymorphous nuclei. PAS stain revealed scant mucin presence. On IHC analysis, the cells stained positively for cytokeratin (CK) 7, 8, 18, 19, and CAM 5.2 and negatively for vimentin, chromogranin (CG), and synaptophysin (SP). They also checked for p53 mutations using p53-CM1 and p53 DO7 antibodies. Results yielded a strong reaction to the p53 DO7 marker, indicating the presence of a p53 mutation, which prompted them to look for KRAS mutations. Thus, Luttges and associates were able to detect a point mutation at codon 12 with a gamma-glutamyl transferase (GGT) to gamma-glutamyl transpeptidase (GAT) switch. As in previously described cases, it was imperative to exclude other primary sites of clear cell carcinomas, notably renal clear cell carcinoma (RCC), which was deemed unlikely due to the absence of vimentin staining. It is noteworthy that they ruled out “sugar” tumors described by Zamboni et al. [14] by the specimens staining negative for human melanoma black (HMB-45). Luttges and colleagues reinforced their argument by proving that the pancreatic head tumor was of ductal origin, not only by the presence of mucin staining but CK 7, 8, 18, and 19 positivity in cells of ductal origin.

In 1998, Radhi and associates reported the case of a family with fibrinogen storage disease, with the father and son developing primary pancreatic cancer [15]. The son died at an early age due to his illness. Microscopy showed cells with “empty-looking cytoplasm” that contained glycogen and stained negative for mucin. The tumor was classified as a clear cell tumor of the pancreas despite the presence of glycogen and lack of mucin.

Case Reports in the New Millennium

In 2004, Sasaki and colleagues described a case of a 61-year-old woman admitted to the hospital for abdominal pain [16]. CT abdomen showed a 14-cm pancreatic body tumor with no metastasis. After resection, she was treated with 5-FU for 14 days. Microscopy showed clear cell nests with abundant clear cytoplasm and centrally/peripherally located nuclei. IHC revealed positive staining for CK 8, 19, and alpha-1 antitrypsin (AAT) and negative staining for CG, SP, and HMB-45. Despite the negative KRAS mutation, they assumed it could still be a variant of ductal carcinoma.

Also in 2004, Ray and colleagues [17] described a case of a 71-year-old man admitted to the hospital for a traumatic fall who was found to have a pancreatic tail tumor. The patient underwent distal pancreatectomy, splenectomy, and partial omentectomy with uneventful postoperative recovery. When grossly examined, the tumor had invaded the splenic artery (but not intraluminally) and surrounded the perineural tissue. Microscopically, the tumor contained abundant clear cells arranged in nests or chords with clear cytoplasm, distinct cell borders, and eccentric nuclei. The cells comprised almost 95% of the tumor. Their belief in the “ductal origin” of the tumor was based on the presence of mucin and positive staining for CK 7, CAM 5.2, CK 20, and CEA and negative staining for SP, CP, vimentin, HMB-45, and CD 10. As with the case described by Luttges et al., Ray and associates detected a missense mutation in codon 12. Although the partial positive staining for NSE and AAT added some skepticism to the ductal nature, the presence of mucin, dense desmoplasia, and perineural invasion features was similar to the ductal features.

A report in 2004 by Batoroev and colleagues discussed a 60-year-old man who was found to have a 4-cm pancreatic head lesion [18]. With the lesion deemed unresectable, the patient was treated with upfront chemotherapy and radiotherapy shortly before expiring. The microscopic assessment showed large and round tumor cells with clear cytoplasm and hyperchromatic nuclei forming irregular nests. They stained positively for PAS and weakly for mucicarmine. IHC revealed positive stains for CEA but negative for vimentin. Electron microscopy demonstrated that the clear cells contained glycogen granules, mucinous droplets, and poorly formed desmosomes in between the cells. Batoroev et al. concluded that this tumor was indeed a ductal adenocarcinoma variant.

More cases followed thereafter, and Ray and colleagues described the case of a 46-year-old man who presented for a pseudocyst removal after suffering from pancreatitis [19]. A hard pancreatic mass and a 14-cm omental mass were sent for pathology. The patient declined treatment and expired shortly thereafter. The authors described seeing large pleomorphic cells with hyperchromatic nuclei and abundant clear cell cytoplasm arranged in tubules on microscopy. Their approach was similar to those adopted by the other authors and focused on ruling out RCC and other differential diagnoses. IHC returned positive for pancytokeratin, CK7, and CEA, and these masses were deemed CCCP.

In 2007, Jamali and colleagues described a 75-year-old man who was found to have a pancreatic head mass [20]. Despite undergoing a successful Whipple procedure, he expired shortly afterward due to liver metastases. The pathologic examination showed that the tumor consisted of three types of malignant cells: squamous, clear cells in a ductal lining, and other large cells with eosinophilic cytoplasm and eccentric nuclei. The clear cells contained glycogen and mucin and stained positive for CK markers, thereby validating their nature. However, how all three aggressive types came to coexist remains a mystery.

Recent Advancements

Prior to 2008, data regarding this entity have been scant, encompassing only a few case reports that described the visualized pathology and IHC findings with hardly any mention of prognostic and therapeutic information due to insufficient data. Kim and colleagues conducted a retrospective cohort analysis that revealed more information regarding CCCP [21]. They reviewed hospital records at a single center institution in Chicago from 2002 to 2008 and searched for all resections of ductal pancreatic carcinoma while excluding intraductal papillary mucinous and endocrine neoplasms. Regardless of the differentiation states, they collected 84 cases that included clinical, histological, and prognostic implications. Kim and associates also focused on detecting the presence of hepatocyte nuclear factor 1 beta (HNF1B), a transcription factor found in these cells that could possibly be mutated and overexpressed. This idea was based on the experience from ovarian clear cell carcinoma and the presence of high hepatocyte nuclear factor-1 beta (HNF1B) levels published by Tsuchiya and colleagues [22], as well as the presence of upregulation in other cancers. Out of the 84, they found 20 cases of tumors containing a clear cell component, 12 of which had more than 75% clear cell involvement, which were called pure clear cell carcinoma. The remaining eight cases had less than 75% clear cell involvement and were referred to as mixed tumors. They described the mixed tumors as having distinct areas of clear cell features within the usual malignant cells forming the ductal structure. The clear cells contained hyperchromatic nuclei, eccentrically or centrally located. Two types of adjacent architectures were noted, one that existed in a duct-like structure that would eventually disperse into a separate nest-like structure. They theorized that this phenomenon was a characteristic of a transitional trend towards dedifferentiation. PAS diastase reaction and mucicarmine staining showed that the intracytoplasmic vacuoles were devoid of glycogen and mucin, but the apices were not.

Kim et al. further expanded their study by looking at clinical profiles. They noticed the absence of significant clinical differences, including age, sex, tumor size, lymph node (LN) involvement, grade, and stage, between the 20 cases versus the conventional 64 cases, with the majority of cases displaying a stage 2B with moderate differentiation. Overall survival among these groups was not statistically significantly different with 56% to 65% mortality (non-clear vs clear). They then stained the samples for HNF1B, which was strongly present in the clear cell variant as opposed to the non-clear variant, which showed mostly a weak pattern of staining. When they compared the stainers with the non-stainers, no significant differences were noticed in tumor grade, LN involvement or stage, but interestingly, worse survival was noted in the stainers’ cohort (i.e. HNF1B+ clear cell type) (P<0.01).

In 2009, Lee and colleagues described a woman with epigastric pain who was diagnosed with metastatic pancreatic cancer to the liver [23]. She received palliative chemotherapy with gemcitabine but expired shortly thereafter. Microscopic examination of the liver lesion revealed findings consistent with large oval cells, well-defined cell membranes, clear cytoplasm, and large nuclei. Nests of these cells formed almost 90% of the tumor. On the other hand, the pancreatic tumor showed rhabdoid cells described as large cells with eccentric nuclei and prominent nucleoli. PAS reaction detected scant mucin inside the cytoplasm with positive staining for pancytokeratin, CK7, CEA, and epithelial membrane antigen (EMA). They hypothesized that the rhabdoid phenotype was a dedifferentiated endpoint, part of the tumor cells’ degeneration phase.

**Figure 2:**
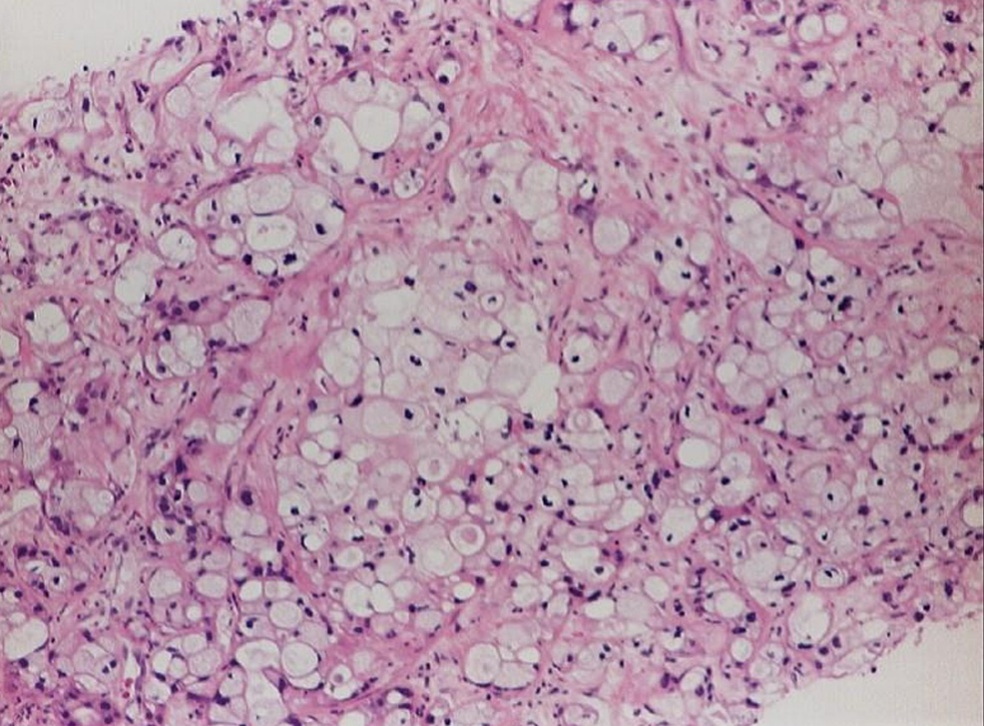
Infiltrating adenocarcinoma showing a clear cell pattern Note: Reprinted from Modi Y, Shaaban H, Gauchan D, Maroules M, Parikh N, Guron G: Primary clear cell ductal adenocarcinoma of the pancreas: a case report and clinicopathologic literature review. J Cancer Res Ther. 2014, 10:773-776. 10.4103/0973-1482.136043 [24]

In 2014, Modi and colleagues described a patient with a pancreatic body/tail mass and liver metastasis [24]. The biopsy of the liver metastasis showed atypical glands composed of pleomorphic cells with distinct cellular borders and clear cytoplasm (Figure 2). In 90% of the tumor, IHC studies revealed positive vimentin, CK7, PAS, and CA19-9. The patient was diagnosed with primary pancreatic clear cell carcinoma. However, despite the positive vimentin, they hypothesized that with the absence of a clear kidney lesion and positive CA19-9 and CEA, CCCP would make more sense.

In a more recent case, Sun and colleagues described a 64-year-old man who was found to have a pancreatic tail mass [25]. After distal pancreatectomy and splenectomy with adjuvant gemcitabine, the patient’s disease progressed with liver metastases. Microscopic examination showed that the tumor contained atypical glands formed of cells with clear cytoplasm, well-defined borders, and pleomorphic eccentric-located nuclei. This composed 90% of the tumor, and the arrangement varied from chords to trabeculae and, in some areas, displayed the hobnail appearance. IHC returned positive for CK7 and negative for HNF1B. They diagnosed the case as CCCP, based on morphology that resembled those of the previous cases described. They acknowledged the role of HNF1B but disputed whether its presence should be absolute.

An interesting study was published in 2018 by Yang and colleagues, whereby they sought to investigate the presence of a marker that could provide an accurate diagnosis of pancreatic adenocarcinoma [26]. They chose HNF1B due to its developmental role during pancreatic morphogenesis and were keen to prove that its expression is present in all types of adenocarcinomas of the pancreas. They reviewed 127 cases of primary pancreatic carcinomas, 17 cases of metastatic pancreatic ductal carcinoma, 47 biliary adenocarcinomas, and 231 pancreatic-like carcinoma cases that were of renal, ovarian, and HCC origin. They noticed that 84% of pancreatic ductal adenocarcinomas (PDAC) stained positive for HNF1B while 94% of the metastatic cases and a rather high percentage among the biliary, kidney, and ovarian stained positive for HNF1B as well. However, HNF1B expression was not associated with overall survival. They also divided PDAC into conventional, mixed, and pure clear cell, and noticed no association between HNF1B staining pattern and intensity and presence/absence of cytoplasmic clearing. Despite this, there were no analyses to evaluate the correlation between staining intensity and overall survival to derive further conclusions. However, they were able to calculate the sensitivity of HNF1B expression, which was 84% with a negative predictive value of 85% and a specificity of 68%.

Finally, O’Neill and colleagues described a 63-year-old man who was found to have a pancreatic neck mass with liver deposits [27]. The liver biopsy showed nests of round cells, large nuclei, clear cytoplasm, and well-defined membranes. IHC was positive for pancytokeratin, HNF1B, and CK7. The patient was treated with one cycle of gemcitabine and nab-paclitaxel after which he clinically improved with pending follow-up.

Discussion

CCCP remains a poorly characterized and understood disease. It is currently listed under “miscellaneous” as part of the World Health Organization (WHO) classification. Our objective was to collect all available data and present a succinct history and characterization of this entity. It has intrigued and yet remained a mystery to all authors. It was very interesting to retrace how the identification and classification of this disease have evolved over time. Cubilla and Fitzgerald were the first to describe a rudimentary picture of primary clear cell tumor of the pancreas, but it was unclear then how and when to classify the disease. As more case reports emerged in the 1980s, the common approach described by the authors was to rule out the presence of RCC, ovarian, or adrenal primary clear cell before assuming it was of pancreatic origin. This made sense at the time, as CCCP was still an ill-described entity, and RCC was known to be a notoriously aggressive disease.

**Figure 3:**
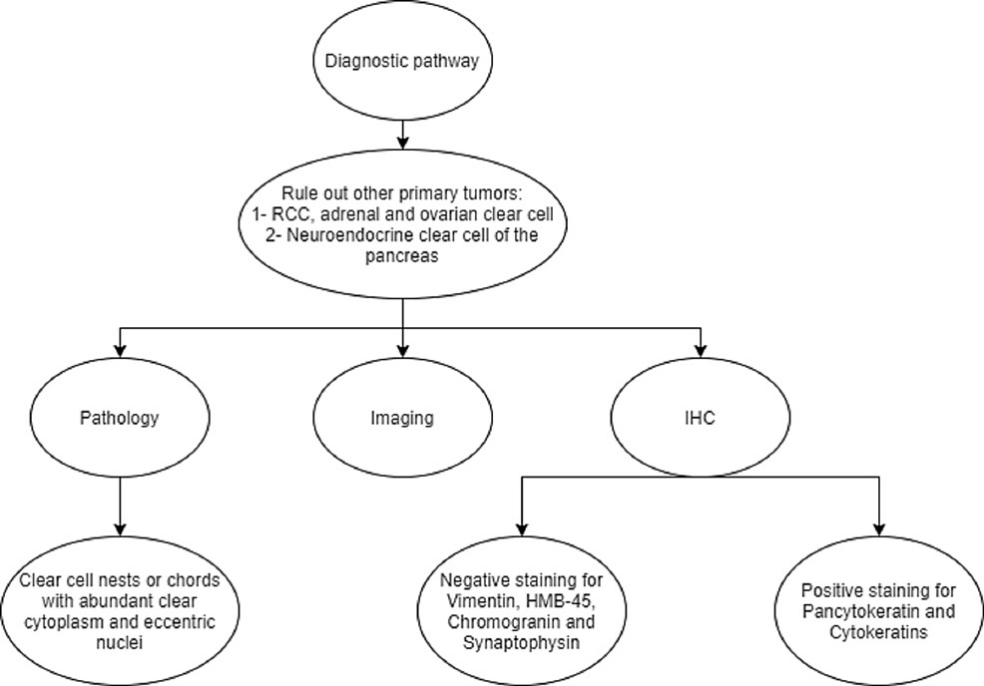
CCCP diagnostic pathway CCCP: clear cell carcinoma of the pancreas

Advancements in detection methods during the 1990s saw the inclusion of IHC as part of the diagnostic arsenal to exclude renal cell and other types of clear cell carcinomas (e.g. negative staining for vimentin that ruled out RCC). Furthermore, IHC and the discovery of certain intracellular structures mostly seen in the pancreatic version of clear cell, such as pancytokeratin and cytokeratins, have facilitated the identification process, as the morphology can be ambiguous (Figure 3). It is important to distinguish CCCP from clear cell neuroendocrine tumors of the pancreas, commonly seen in patients with von Hippel-Lindau. Clear cell neuroendocrine tumor of the pancreas has a similar histologic appearance (clear cell cytoplasm and prominent nuclei), but with IHC stains positive for CG and SP [28-29]. With subsequent authors reporting certain positively staining features, this has given the disease some character and identity. Moreover, the presence of KRAS mutations and p53 has further steered the suspicion towards a ductal origin, as more than 90% of PDAC have been shown to have a KRAS mutation. Most of the authors have commented that the tumor cells existed in nests or chords, and in some cases, they noticed the presence of both architectural variants as a sort of transitional phase. Sometimes, within these nests or chords themselves, they saw a transition between the type of tumor cells, such as clear cells and rhabdoid cells, indicating a possible dedifferentiation phase [20]. More interesting is the presence of a combination of tumor cells within the primary pancreatic lesion, but the dominance of the clear cell type in the metastatic lesion [11,20]. This might suggest the possible aggressiveness of the clear cell type compared to other neighboring neoplastic cell types.

As in PDAC, multiple patients with CCCP succumb to their disease, often over a short period of time. The tumor can arise from the body [11-12,16], head [13,15,18-20,30], tail [17,23-25], or even the neck of the pancreas [27]. There are other cases that presented with metastasis at diagnosis [11-12,19,23-25,27]. Most of the authors have described treating CCCP in the same manner as PDAC would have been treated in each time period. Thus, the change in treatment modalities mimics that of PDAC, with patients in the 1980s and 1990s receiving 5-FU, Mitomycin C, and surgical resection. A transition to gemcitabine was observed in the more recent cases. Although some patients were smokers and had a history of drinking alcohol, the information needed to derive any conclusions from these modifiable risk factors remains poor. It remains unknown how current PDAC therapies impact CCCP, but in the most recent case report described, we see that the patient has responded well to gemcitabine and nab-paclitaxel and will be followed up in the future [27].

Although the morphological classification and definition remain of paramount importance, there is no denying that the molecular biology of a tumor is a complementary and equally important aspect, especially how it pertains to therapeutic options. HNF1B is known as one of the transcription factors needed for the early development of internal organs such as the kidneys, pancreas, and liver [31]. A mutation in HNF1B is one of the causes of pancreatic agenesis or hypoplasia [32]. HNF1B overexpression has been noted in several other tumors, including ovarian clear cell carcinoma (CCC) [22], clear cell adenocarcinoma of the bladder [33], prostate cancer [34], and endometrial cancer [35]. Tsuchiya and colleagues hypothesized that ovarian CCC might be a wholly distinct entity apart from epithelial ovarian carcinoma and that HNF1B over-expression in these cancers was a contributing factor [21]. They noticed that by reducing the expression of HNF1B in these tumor cells in vitro, apoptosis was induced and led to cell death, thereby suggesting that HNF1B is required for cell survival. They also suggested the possibility of molecular targeted therapy for ovarian CCC, which would not be unreasonable seeing that ovarian CCC is now known to be extremely chemo-resistant, especially to platinum-based chemotherapy [36]. Strong positivity in HNF1B staining in CCCP, as opposed to PDAC cases, was demonstrated by Kim and associates [21], and strong staining for HNF1B indicated a worse overall survival versus tumors with weak staining. This would further raise the possibility of whether CCCP, as with ovarian CCC, is a slightly distinct entity with more chemo-resistance. In a recent study by Nie and colleagues, they noticed that positive HNF1B staining was found to be negatively associated with overall survival in pancreatic ductal adenocarcinoma (HR=1.54 and P=0.038) [35]. They also showed an association between HNF1B expression and immune cell infiltration into some tumors, indicating active tumoral immune involvement. All these studies demonstrate the importance of molecular studies in stratifying disease responsiveness, prognosis, and expected clinical outcome and may provide further insight into directing targeted therapies as in the non-small cell lung cancer experience.

Table 1 summarizes the cases described in all the CCCP case reports discussed so far.

**Table 1:** Clinical characteristics and outcomes of the cases described in all the CCCP case reports published Note: U refers to unspecified CCCP: clear cell carcinoma of the pancreas; CEA: carcinoembryonic antigen; CA19-9: carbohydrate antigen 19-9; HMB-45: human melanoma black; 5-FU: fluorouracil; CK: cytokeratin

Author and year	Age	Sex	Presenting Symptom	Smoking History	Alcohol	Family History	Markers	Tumor Site in Pancreas	Pathology	IHC	Mets	Treatment	Outcome
Urbanski et al. 1982 [11]	57	M	Epigastric pain	U	U	U	U	Body	Large cells with clear cytoplasm and hyperchromatic nuclei	U	Liver	None	Expired 6 weeks post-diagnosis
Kanai et al. 1987 [12]	71	M	Abdominal pain	None	None	None	CEA 628 ng/ml CA 19-9 9900 U/ml	Body	Large bulky oval cells with abundant intracytoplasmic vacuoles and eccentric nuclei	U	Lungs	Mitomycin C and 5-FU	Expired 51 days post-diagnosis
Luttges et al. 1997 [13]	53	M	Abdominal pain	U	U	U	CA19-9 90.3ng/ml	Head	Large cells with large nuclei and abundant clear cytoplasm	Positive: CK 7,8,18,19 Negative: Vimentin; chromogranin; synaptophysin	Liver (11 months post-diagnosis)	Partial duodenopancreatectomy	U
Radhi et al. 1997 [15]	37	M	Abdominal pain	U	U	Father	U	Head	Cells with empty-looking cytoplasm, small nuclei arranged in a pseudoacinar manner	Positive: low molecular weight keratin; CEA; epithelial membrane antigen Negative: c-erbB-2	none	Cholecystojejunostomy	Expired post-surgery
Ray et al. 2004 [17]	75	M	Abdominal pain	U	U	U	U	Tail	Pleomorphic cells with abundant clear cytoplasm	Positive: CK 7, 20; CAM 5.2 Negative: Vimentin; Synaptophysin; Chromogranin; HMB-45	none	Distal pancreatectomy Splenectomy Partial omentectomy	U
Sasaki et al. 2004 [16]	61	F	Abdominal pain	U	U	U	CA19-9 44 u/ml; CEA 1.1 ng/ml	Body	Clear cell nests, with abundant clear cytoplasm, eccentric nuclei	Positive: CK 8,19 Negative: Chromogranin; synaptophysin; HMB-45	none	Pancreatectomy and 5-FU	U
Batoroev et al. 2004 [18]	60	M	Abdominal pain	U	U	U	U	Head	Abundant clear cytoplasm with pleomorphic nuclei	Negative: Vimentin	U	Adjuvant radio and chemotherapy	Expired 4 months post-diagnosis
Ray et al. 2005 [19]	46	M	Abdominal pain	20	+	U	CEA 2 u/ml CA 19-9 2 u/ml	Head	Abundant clear cytoplasm with large nuclei	Positive: Pancytokeratin; CK 7 Negative: HMB-45; Vimentin; Chromogranin; Synaptophysin	Omentum	Patient refused	Expired 3 months post-diagnosis
Jamali et al. 2007 [20]	75	M	Abdominal pain	35	+	Non-contributory	U	Head	Large clear cells with pleomorphic nucleoli	Positive: Cytokeratin; Vimentin	none	Whipple and Chemotherapy	Expired 6 months post-diagnosis
Lee et al. 2009 [23]	66	F	Abdominal pain	No	No	Non- contributory	CEA 12.17 ng/ml CA 19-9 597.1 U/ml	Tail	Oval cells with well-defined borders, large nuclei, and clear cytoplasm	Positive: CK 7 Negative: Chromogranin; Synaptophysin; HMB-45	Liver	Gemcitabine	Expired 1-month post-diagnosis
Modi et al. 2014 [24]	74	F	Abdominal pain	U	U	U	CEA 600 u/ml CA 19-9 7000 u/ml	Tail	Abundant clear cell with well-defined cell boundaries	Positive: CK 7; Vimentin Negative: CK 20; Chromogranin; synaptophysin; HMB-45	Liver	none	U
Ahls et al. 2014 [30]	43	F	Abdominal pain	No	No	U	U	Head	Oval cells with abundant clear cytoplasm and large nuclei	Positive: Pancytokeratin; CK 7, 8/18 Negative: Synaptophysin; Chromogranin; HMB-45	none	Pyloris preserving pancreatoduodenectomy	U
Sun et al. 2018 [25]	64	M	Abdominal pain	30	+	Non- contributory	CEA 18.56 ng/ml CA 19-9 649.15 U/ml CA 125 133 U/l CA 242 394	Tail	Oval bulky cells with abundant clear cytoplasm	Positive: CK 7 Negative: Chromogranin; synaptophysin	Liver	Resection and Gemcitabine	Expired 2 months post-diagnosis
O’ Neill et al. 2020 [27]	63	F	Abdominal pain	U	U	Non- contributory	CEA 4 ug/l CA 19-9 916 ku/l CA 125 235 ku/l	Neck	Oval cells, large nuclei, clear cytoplasm	Positive: Pancytokeratin; CK 7 Negative: HMB-45; Chromogranin; Synaptophysin	Liver	Nab-paclitaxel and Gemcitabine	U

## Conclusions

As with any disease, the characterization and identity of CCCP evolved with time in parallel to the advancements witnessed in science. With the help of new technologies, what was once vague and diffuse has become more defined. The diagnosis of this entity requires histologic evidence of a clear cell cytoplasm with an eccentric nucleus, positive IHC stains for pancytokeratin and other cytokeratins such as CK7, in addition to ruling out other pathologies such as neuroendocrine tumors and RCC with a negative stain for CG, SP, and Vimentin, respectively. The treatment algorithm remains similar to PDAC; however, the discovery of the molecular marker HNF1B might have a prognostic and therapeutic significance in the future. We attempted to provide further insight into a poorly understood entity, indirectly and simultaneously portraying how scientific methods of detection and diagnosis mature with time until specific diagnoses can be made and targeted therapy can be established.
